# CD3e expression in HTLV-1-infected individuals is associated with proviral load and Tax expression

**DOI:** 10.1186/1742-4690-8-S1-A114

**Published:** 2011-06-06

**Authors:** Mariana Tomazini Pinto, Tathiane Maistro Malta, Daniel Guariz Pinheiro, Evandra Strazza Rodrigues, Rodrigo A Panepucci, Alessandra P Souza, Kelen C R Malmegrim, Patrícia V B Palma, Osvaldo M Takayanagui, Dimas Tadeu Covas, Simone Kashima

**Affiliations:** 1 Regional Blood Center of Ribeirão Preto, Ribeirão Preto, Brazil; 2 Faculty of Pharmaceutical Sciences of Ribeirão Preto, University of São Paulo, Ribeirão Preto, Brazil; 3 Faculty of Medicine of Ribeirão Preto, University of São Paulo, Ribeirão Preto, Brazil

## Introduction

CD4+ T cells play a central role in HTLV-1 infection. We investigated the global gene expression profile of circulating CD4+ T cells in distinct clinical status of HTLV-1-infected individuals in regard to Tax expression levels.

## Methods

The microarray platform used 12 individual samples divided according to patients’ clinical status and Tax expression as follows: healthy control (CT, n=4), HAC (n=4, 2 high Tax expression and 2 low Tax expression) and HAM/TSP group (n=4, 2 high Tax expression and 2 low Tax expression). Proviral load (PVL) was quantified by qRT-PCR and Tax expression was analyzed by flow cytometry in HAC and HAM/TSP group.

## Results

Hierarchical clustering analysis showed that CT and HTLV-infected groups clustered separately. We also observed that HAC and HAM/TSP groups were in separate clusters regardless Tax expression. We identified 449 genes differentially expressed between HAC and HAM/TSP groups and we classified these genes according the biological functions. CD3e was represented in many functions like cellular development, cell signaling, and others. CD3e expression by qRT-PCR was higher (1.3X) in the HAM/TSP than HAC group (p=0.0195). We also validated LCK, VAV and ZAP70 genes, which are downstream molecules of the CD3e activation pathway. These genes were also significantly higher in HAM/TSP group and CD3e, LCK and VAV1 genes were positively correlated with PVL and Tax expression.

## Conclusion

The higher PVL and Tax expression the higher activity of CD4+ T cells in the symptomatic group, suggesting that this pathway could have an important role in HAM/TSP development.

